# Synthesis of a novel porous Ag_2_O nanomaterial on ion exchange resin and its application for COD determination of high salinity water

**DOI:** 10.1038/s41598-021-91004-w

**Published:** 2021-06-01

**Authors:** Trung Thanh Nguyen, Quynh Anh Nguyen Thi, Ngoc Hang Le, Nhat Huy Nguyen

**Affiliations:** 1 Laboratory of Nanomaterial, An Giang University, 18 Ung Van Khiem Street, Dong Xuyen Ward, Long Xuyen City, An Giang Province Vietnam; 2 Vietnam National University Ho Chi Minh City, Linh Trung Ward, Thu Duc District, Ho Chi Minh City, Vietnam; 3 Faculty of Environment and Natural Resources, Ho Chi Minh City University of Technology (HCMUT), 268 Ly Thuong Kiet St., Dist. 10, Ho Chi Minh City, Vietnam

**Keywords:** Environmental sciences, Chemistry, Materials science, Nanoscience and technology

## Abstract

This study reports for the first time on the synthesis of novel resin@P-Ag_2_O material and its application for reducing the chloride effect on COD determination of high salinity water. This engineered core–shell nanomaterial with cationic ion exchange resin core and porous Ag_2_O shell was prepared by facile ion exchange and silver oxidation method at ambient temperature without using toxic chemicals. The material was characterized by FTIR, XRD, SEM, and SEM–EDX mapping. In the chloride removal test, this material gave a high adsorption capacity of ca. 244 mgCl/gAg at the mild condition with high durability after several adsorption–desorption cycles. Moreover, resin@P-Ag_2_O was applied for removing chloride in water to improve the accuracy of the SMEWW 5220C:2012 method for COD determination of high salinity water. The result showed that the COD of a water sample with salt content after being treated by the material had a low error (≤ 10%) as compared to the sample without salt. Meanwhile, the COD of salty water measured by the dilution method had an error of around 15%. These results indicate that resin@P-Ag_2_O material has a very potential application for chloride removal and COD determination of high salinity water.

## Introduction

Chemical oxidation demand (COD) is a very popular and important parameter for evaluating the organic pollution level of water sources as well as for designing and constructing wastewater treatment systems. COD value of a water sample is determined by the SMEWW 5220C:2012 method^1^, where the sample was treated with dichromate under the acid condition and Ag^+^ catalyst at 150 °C for 2 h. The reactions during COD determination using potassium hydrogen phthalate (KHP, used as a COD standard) follow Eqs. (1) and (2), where one mole of Cr_2_O_7_^2−^ consumed for the oxidation of KHP corresponds to 1.5 mol of oxygen^1,2^.

1 $$2K{C}_{8}{H}_{5}{O}_{4}+10{K}_{2}{Cr}_{2}{O}_{7}+41{H}_{2}{SO}_{4} \to 16{CO}_{2}+46{H}_{2}O+10{Cr}_{2}{\left({SO}_{4}\right)}_{3}+11{K}_{2}{SO}_{4}$$

2 $$2K{C}_{8}{H}_{5}{O}_{4}+15{O}_{2}+{H}_{2}{SO}_{4} \to 16{CO}_{2}+6{H}_{2}O+{K}_{2}{SO}_{4}$$

For water samples (both surface water and wastewater) with high chloride (i.e. > 1.0 g/L), the SMEWW 5220C:2012 method has a positive deviation due to the reactions presented in Eqs. (3) and (4)^1,2^. In order to reduce the error, a certain amount of HgSO_4_ is added for forming the HgCl_2_ complex. However, due to its high toxicity, the use of mercury is applied with caution.

3 $$6{Cl}^{-}+{C{r}_{2}{O}_{7}}^{2-}+{14H}^{+} \to {2Cr}^{3+}+3C{l}_{2}+7{H}_{2}O$$

4 $$2N{H}_{4}^{+}+3C{l}_{2} \to {N}_{2}+6HCl+2{H}^{+}$$

Three different approaches (i.e. new, corrected, and modified methods) have been proposed for improving the COD measurement of highly salty water^3^. The new methods were developed to reduce the time for determination such as the chemiluminescence system to analyze Cr^3+^ instead of Cr^6+^^4^ and the flow-injection method using KMnO_4_ and ion exchange column to collect and analyze Mn^2+^^5^ or Ce(SO_4_)_2_ and analyze Ce^4+^^6^. Other techniques used new digestion methods such as microwave- and ultrasound-assisted digestions^7,8^ and their combination with using Mn(III)^9^ and Ce(IV)^6^ as oxidants for faster and more complete oxidation. Although these new methods have a short determination time with high sensitivity, they require new instruments and new techniques. In correction methods, the real COD from organics was determined by the subtraction of apparent COD to the COD by chloride. This method is labor-intensive with several steps of (i) measure the chloride concentration, (ii) prepare the synthetic water sample with the same chloride but without organics content, (iii) measure the COD values of both actual and synthetic samples, and (iv) subtraction for obtaining the real COD value. Therefore, modification based on the current method is considered a preferable choice for practical applications among the three approaches.

Several modification methods have been proposed for reducing the error of COD determination of highly salty water such as sample dilution, increase of HgSO_4_/Cl^−^ ratio, using masking agents, decrease of K_2_Cr_2_O_7_ amount, applying of new digestion methods^3^. In the dilution method, a water sample is added with water to decrease the chloride concentration to under 2.0 g/L without masking agents and the deviation of this method is in the range of 0.15–5.8% due to dilution error^10^. This method is suitable for water samples with high contents of both organic compounds and chloride. However, it has low accuracy for water with high chloride but low COD value. The use of a high HgSO_4_:Cl^−^ ratio (e.g. 20:1) is also applied for the sample with high chloride but low organic content with a deviation of < 10% and the deviation could reach 12% for the sample with a NaCl concentration of 40 g/L^11^. However, this method is used with caution due to the high toxicity of mercury compounds as well as this method does not completely eliminate the effect of chloride. In the masking method, masking agents such as Ag^+^^12^, Cr^3+^^13^, and Al^3+^^14^ are added for reducing free chloride, where silver nitrate is considered as the most suitable one for precipitation of free chloride ion in AgCl form in water with Cl^−^:COD ratio of ≤ 3:1. However, this method is not suitable for the water sample with Cl^−^:COD ratio of > 3:1. Besides, the pollution of the masking agents in water or its recovery has not been addressed. Since the reaction of K_2_Cr_2_O_7_ with organics is faster than that with chloride, decrease in K_2_Cr_2_O_7_ content^11^ and/or combine it with the increase of HgSO_4_:Cl^−^ ratio^15^ could reduce the effect of chloride. This method is complex since the suitable content of K_2_Cr_2_O_7_ and ratio of HgSO_4_:Cl^−^ need to be determined. Another method is utilizing Bi-based adsorbent to adsorb the HCl produced during the acidification of chloride by 18 N H_2_SO_4_ solution to obtain a chloride-free sample before COD analysis^2^. The process uses concentrate H_2_SO_4_ solution at 150 °C as well as BiOOH material to absorb HCl, which seems not to be an environmental-friendly method. Although proposed and tested by several studies, these approaches have their disadvantages, thus finding a new, effective, cheap, and facile method for improving the accuracy of COD determination is necessary for water samples with a sodium chloride concentration of over 2 g/L.

This study proposes a new and facile solution by using a specially designed porous Ag_2_O shell on cationic exchange resin core (resin@P-Ag_2_O) for selective removal of the chloride content before COD measurement of the salty water sample. The material was synthesized by simple ion exchange and silver oxidation method at room temperature without using toxic chemicals. By using this material, the determination of COD for salty water is simple, highly accurate, environmental-friendly, and low-cost due to the recyclability of the material.

## Experimental section

### Chemicals and materials

All analytical grade chemicals such as AgNO_3_, NaOH, NaCl, K_2_Cr_2_O_7_, and Fe_2_(SO_4_)_3_ reagents were from Merck (Germany) and directly used without further purification. Standard potassium hydrogen phthalate (KHP) solution was received from Hach (USA). Deionized (DI) water was from a Millipore Milli-Q water system in the laboratory using double-distilled water as the inlet water source. Macroporous Purolite C145 cation exchanger with sulfonic acid (SO_3_^−^) functional groups shipped in Na^+^ form was kindly obtained from Purolite (China) with properties as described in Table S1 of Supplementary Information.

### Generation of resin@P-Ag_2_O material

The synthesis of resin@P-Ag_2_O material was conducted in 2 steps. At first, polymeric macroporous cation exchange resin with strong acid functional groups was preloaded with Ag^+^ cation that is substantially insoluble (Eq. 5). After that, Ag_2_O within the pore phase of macroporous cation exchanger and the porous shell was synthesized by soaking of $\overline{\left(RS{O}_{3}^{-}\right){Ag}^{+}}$ in NaOH solution (Eq. 6).

5 $$\overline {\left( {RSO_3^ - } \right)N{a^ + }} + Ag_{(aq)}^ + \leftrightarrow \overline {\left( {RSO_3^ - } \right)A{g^ + }} + Na_{(aq)}^ +$$

6 $$\overline{2\left(RS{O}_{3}^{-}\right){Ag}^{+}}+2NaOH\to \overline{2\left(RS{O}_{3}^{-}\right){Na}^{+}}+A{g}_{2}{O}_{(s)}+{H}_{2}O$$

In a typical procedure, 30 g of ion exchange resin was added into 200 mL of 4% AgNO_3_ solution at pH 6 and the mixture was stirred for 3 h. The exchanged resin was then collected and preliminarily washed with DI water. After that, the washed resin was added into 200 mL of 10% NaOH solution and stirred for 4 h in order to form Ag_2_O nanoparticles in the pore and the porous shell on the resin. The material (resin@P-Ag_2_O) was then collected and washed with DI water several times and finally dried at ambient temperature naturally. Materials with different Ag loadings were synthesized by using AgNO_3_ solution with different concentrations.

### Material characterization

Silver loading was determined by using an inductively coupled plasma optical emission spectrometer (ICP-OES, Optima 20000DV, Perkin Elmer). Before analysis, the sample was treated with a 2% HNO_3_ solution to ensure all non-particle forms of silver are dissolved and removed. The surface chemical property of resin@P-Ag_2_O was carried out using Fourier transform infrared spectroscopy (Bruker-FTIR, 500–4000 cm^−1^). Wide-angle X-ray diffraction (WAXRD) patterns were recorded using a D2 Phaser XRD 300 W diffractometer equipped with CuKα radiation (λ = 1.5406 Å) at step size and step time of 0.01° and 30 s, respectively. The morphology, particle size, and elemental dispersion of the resin@P-Ag_2_O sample were evaluated by scanning electron microscopy (SEM) and SEM–EDX mapping techniques. Additionally, the average composition of the resin@P-Ag_2_O sample was also determined by energy-dispersive X-ray spectroscopy (EDX).

### Batch adsorption experiment

In the chloride adsorption test, 0.5 g of the adsorbent was added into 50 mL of 4 g/L chloride solution and the mixture was shaken in a thermostatic water-bath shaker operated at speed of 120 rpm. After reached equilibrium condition, the adsorbent was collected by filtration, and chloride concentration in the supernatant was analyzed by conductivity determination of chloride solution (SPECORD 210 Plus, Analytik Jena, Germany). The used resin@P-Ag_2_O material was then regenerated by dipping into a 5% NaOH solution for 45 min. After regenerated, the adsorbent was rinsed with DI water several times before applying for chloride removal in the next test cycle. All experiments were replicated three times and the average results were reported in this study. The adsorption removal efficiency and adsorption capacity (defined as the amount of chloride adsorbed per mass unit of adsorbent) were calculated from the following equations:

7 $$H = \frac{{C_{o} - C_{t} }}{{C_{o} }} \times 100\%$$

8 $$q_{e} = \frac{{C_{o} - C_{e} }}{m} \times V$$

where: qe (mg/g) is the adsorption capacity and H (%) is the removal efficiency. C_o_, C_t_, and C_e_ (mg/L) are the initial, at time t, and equilibrium chloride concentrations in the aqueous solution, respectively. V (L) is the volume of the solution and m (g) is the weight of silver content.

### Application of resin@P-Ag_2_O for COD analysis

KHP stock solution was diluted with DI water to form a COD standard solution of 120 mgCOD/L. NaCl was then added into the standard solution to form a series of 100-mL samples with NaCl concentration in a range of 0, 1.0, 1.5, 2.0, and 2.5 g/L. In the modified COD test, before obtaining the suitable condition, preliminary experiments were done by adding 2.0 g of resin@P-Ag_2_O into 100 mL of a standard solution containing NaCl for 30 min of adsorption. The material was then separated and the solution was taken for COD analysis followed the conventional SMEWW 5220C:2012 method.

## Results and discussion

### Material synthesis and characterization

**Figure 1:**
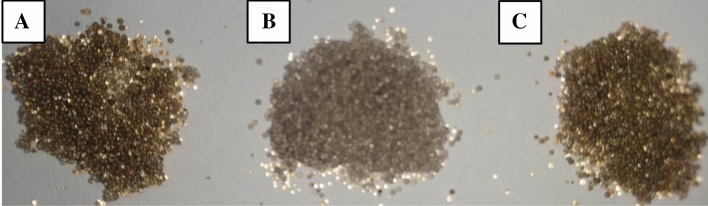
Photos of (**A**) fresh, (**B**) used, (**C**) regenerated resin@P-Ag_2_O materials.

The photos of resin@P-Ag_2_O samples are shown in Fig. 1, where the particle size of the synthesized material is determined to be around 0.5 mm. It can be observed that colors of fresh (Fig. 1A), chloride-adsorbed (Fig. 1B), and regenerated (Fig. 1C) resin@P-Ag_2_O are black, grey, and black colors, respectively. The chloride adsorption turns the black color of resin@P-Ag_2_O into a grey color, indicating the adsorption of chloride on the surface of Ag_2_O particles. Later, treatment with NaOH recovers chloride-adsorbed resin@P-Ag_2_O in grey color back to its fresh condition with black color, implying the successful regeneration of the material.

**Figure 2:**
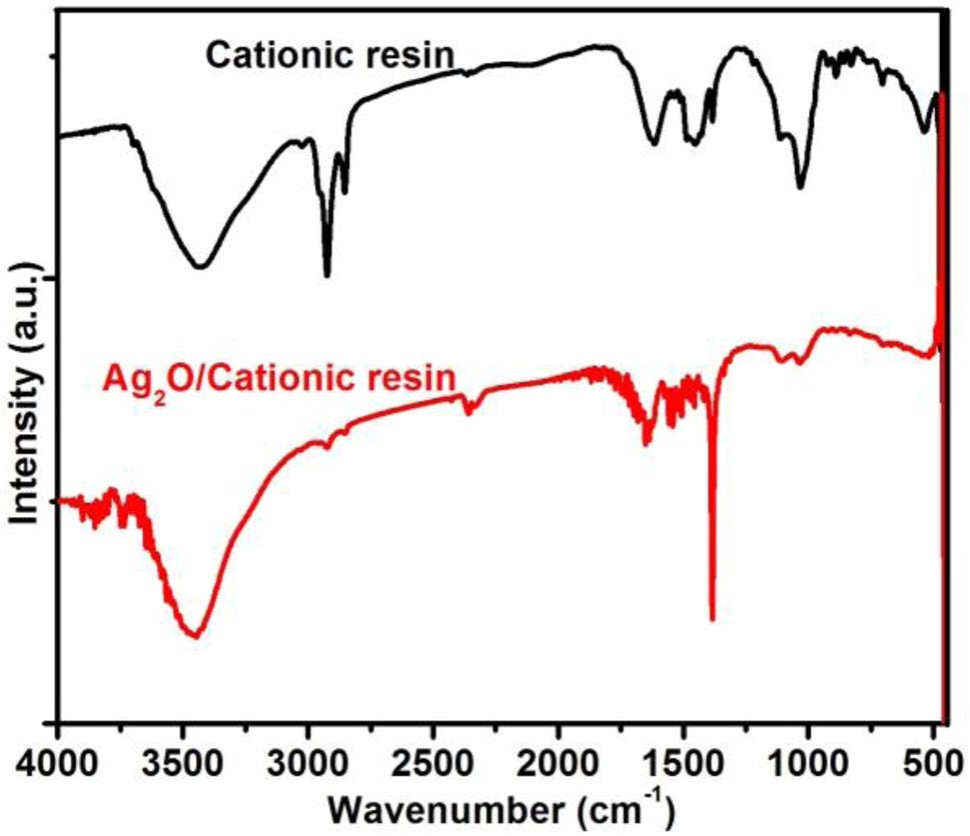
FTIR patterns of strong-acidic cationic resin and resin@P-Ag_2_O materials.

FTIR was applied for identifying the chemical composition in the structure of cationic exchange resin (Purolite C145) and resin@P-Ag_2_O, as shown in Fig. 2. For cationic exchange resin, the FTIR peaks in the wavenumber range of 3360–3590 cm^–1^ are simple O–H bond with the highest peak at 3457 cm^–1^ while the wavenumber range of 2800–3060 cm^–1^ is specific to various bonds in polystyrene structure with a peak at 2922 cm^–1^. The peak at 1601 cm^–1^ is specific for the C–C simple bond of the styrene ring while peaks at 1039, 1128, and 1181 cm^–1^ are attributed to the sulfonic group of SO_3_. The peak at 835 cm^–1^ confirms the substitution at the benzene ring level by sulfonic groups and divinylbenzene cross-linking. For FITR of resin@P-Ag_2_O material, its FTIR pattern is similar to that of cationic exchange resin except for a high-intensity peak at 1300 cm^−1^ of Ag_2_O vibration.

**Figure 3:**
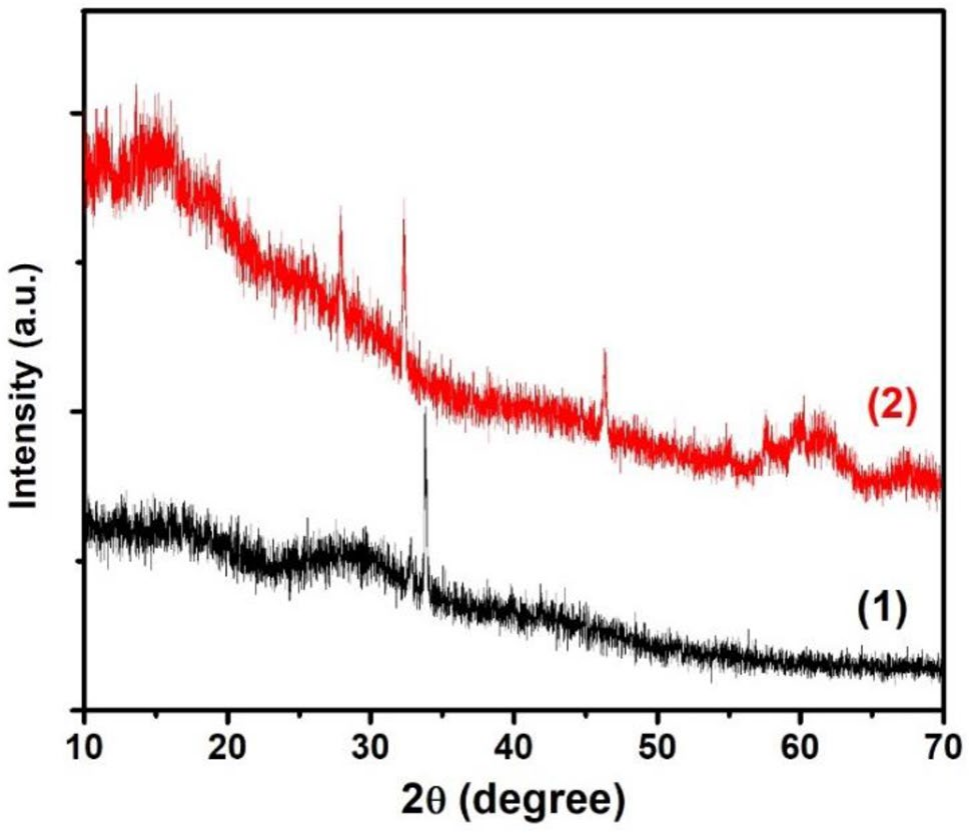
XRD patterns of (1) resin@P-Ag_2_O and (2) Cl-resin@P-Ag_2_O materials.

XRD patterns of fresh resin@P-Ag_2_O and resin@P-Ag_2_O after chloride adsorption (Cl-resin@P-Ag_2_O) are plotted in Fig. 3. For resin@P-Ag_2_O material, the highest peak of Ag_2_O observed at 2θ of 33.5° is attributed to as-synthesized Ag_2_O at the facet of (111). This confirms the successful synthesis of resin@P-Ag_2_O by the proposed protocol of the facile ion exchange and silver oxidation method. In Cl-resin@P-Ag_2_O material, the XRD peaks of 28°, 32°, and 46° are attributed to the presence of AgCl (111), AgCl (200), and AgCl (220), respectively, implying that crystalline AgCl formed with the grey color is the result from chloride adsorption using resin@P-Ag_2_O.

**Figure 4:**
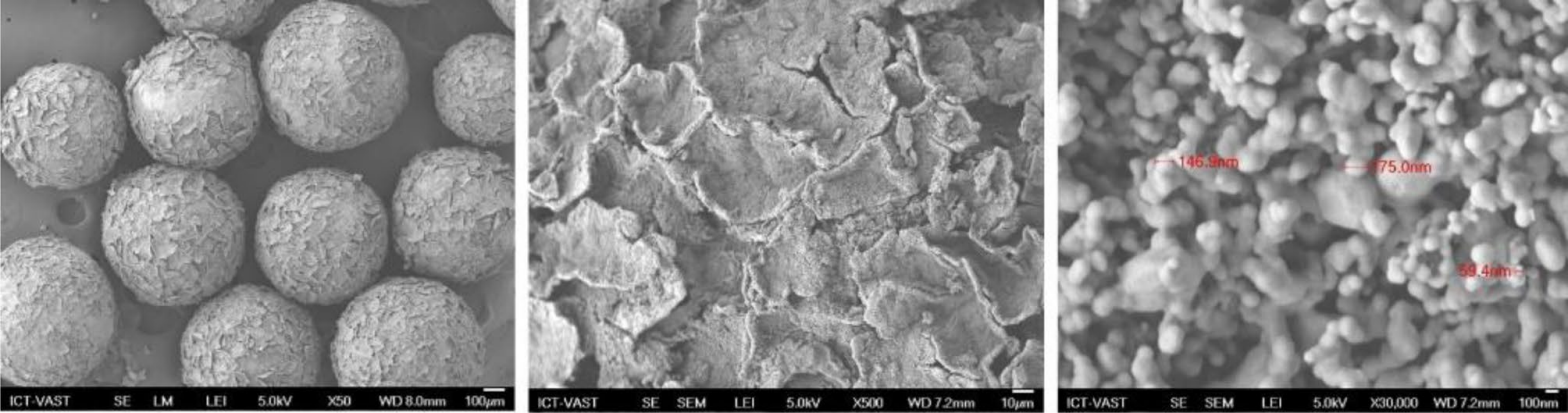
SEM images of resin@ P-Ag_2_O material at different resolutions.

**Figure 5:**
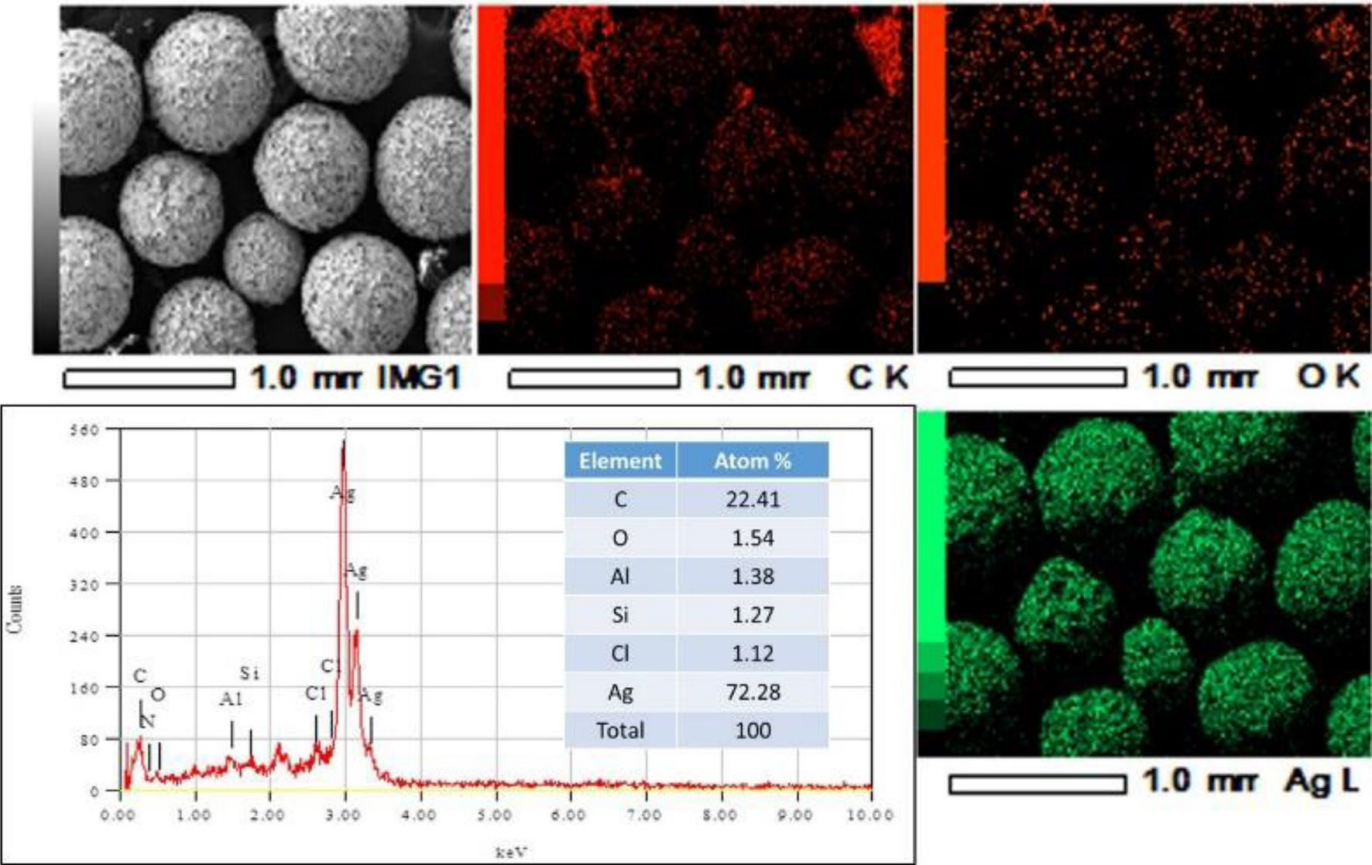
SEM–EDX mapping analysis of resin@ P-Ag_2_O material.

**Figure 6:**
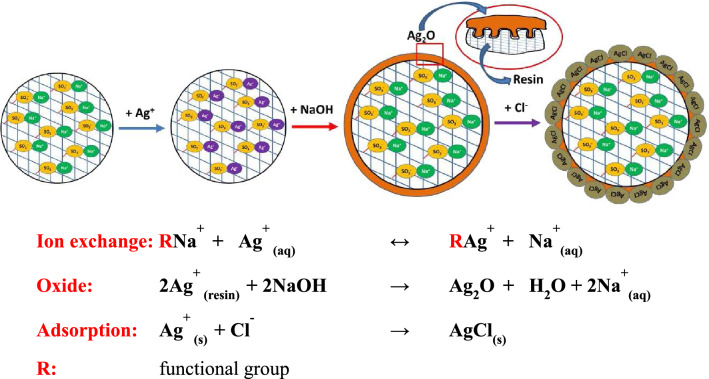
Concept of resin@P-Ag_2_O synthesis and its application for chloride removal.

The SEM images of resin@P-Ag_2_O material at different resolutions and its SEM–EDX mapping results are displayed in Figs. 4 and 5, respectively. Obviously, resin@P-Ag_2_O has a spherical shape from the hard framework of the cationic exchange resin core. Additionally, the results from Fig. 5 confirm that the shell of the material is mainly composed of Ag_2_O with an atomic percentage of 72.28%. As also seen in Fig. 4, SEM images with high resolution confirm that as-synthesized Ag_2_O on the cationic resin surface is in an array form and a porous structure with nanosize Ag_2_O particles. Based on the material characteristics, the mechanism for the formation of resin@P-Ag_2_O material and its application for chloride removal is summarized and illustrated in Fig. 6.

### Chloride removal experiments

**Figure 7:**
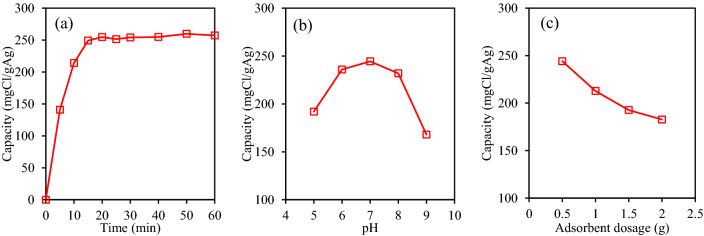
Effect of (**a**) adsorption time (dosage 0.5 g, 4.0 gCl/L, pH 7), (**b**) solution pH, and (**c**) adsorbent dosage on chloride removal.

The chloride adsorption of resin@P-Ag_2_O during 120 min of the experiment is illustrated in Fig. 7a, proving the rapid adsorption of chloride was obtained with fast equilibrium reached after 20 min. The experimental condition was then chosen at 20 min of equilibrium time and 4.0 gCl/L of chloride concentration (i.e. similar to brackish water). The effect of solution pH (adjusted by using HNO_3_ or NaOH solution) was investigated in a range of 5 to 9 (as found in natural surface water) and the result is presented in Fig. 7b. It is noted that the adsorption capacity is relatively similar at 230 to 244 mgCl/gAg in the pH range of 6 to 8, which is very suitable for the practical surface water sample application. However, the capacity was lower at pH 5 and pH 9 with values of 192 and 168 mgCl/gAg, respectively, suggesting low or high pH value is not favorable for chloride adsorption. At pH 5, a part of dissociated chloride ions (Cl^−^) could be acidified in the form of HCl, and its potential to form AgCl on the resin@P-Ag_2_O surface is slightly reduced. At pH 9, hydroxide ion (OH^−^) competes with Cl^−^ for adsorption on the surface of resin@P-Ag_2_O. Figure 7c describes the effect of adsorbent dosage on the chloride removal in a range of 0.5–2.0 g in 50 mL of 4.0 g/L of chloride solution. As expected, the adsorption capacity decreased with the increase of adsorbent dosage, and the dosage was chosen at 1.0 g/50 mL, which is suitable for practical applications. In summary, the suitable condition for chloride adsorption in 50 mL of 4 g/L was pH 6–8, adsorbent amount of 1.0 g, and after 20 min of equilibrium time.

**Figure 8:**
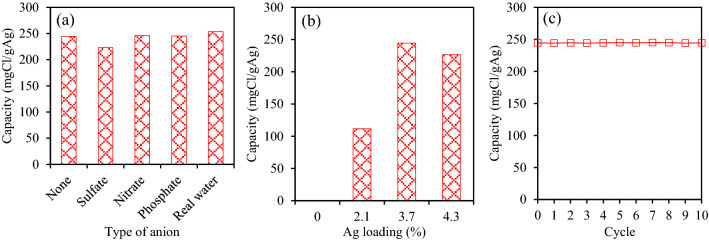
Chloride adsorption capacities under the effects of (**a**) other anions and (**b**) Ag loading and (**c**) stability for chloride removal of resin@P-Ag_2_O material.

It is widely accepted that the presence of anions such as sulfate, nitrate, and phosphate may have strong effects on the adsorption of chloride ions using cation exchange resin^16^. In this study, the effect of these anions was examined at a concentration of 500 mg/L for each anion. The real salinity water (taken at 3 km from the Co Chien river estuary) was also tested for investigation of the simultaneous presence of these ions in the actual water environment. Results in Fig. 8a showed a very little effect of nitrate and phosphate on the chloride adsorption using resin@P-Ag_2_O, possibly due to the highly selective adsorption of chloride on Ag_2_O in terms of thermodynamic and kinetic. However, the chloride adsorption capacity decreased around 8% under the presence of sulfate, which could attribute to the competition of sulfate for forming Ag_2_SO_4_ precipitate on the surface of resin@P-Ag_2_O material. Interestingly, the adsorption capacity increased around 3% in the case of a real water sample via an unclear mechanism that needs further in-depth investigation.

The effect of Ag loading on the chloride adsorption is demonstrated in Fig. 8b, where Ag content (wt.%) increases from 0 (original resin) to 2.1, 3.7, and 4.3 (resin@P-Ag_2_O). As seen in Fig. 8b, pure resin does not adsorb any chloride due to its cationic characteristics. The adsorption capacity increased when Ag loading increased from 2.1 to 3.7, where it reaches the highest capacity due to the sufficient amount and effective dispersion of Ag on the surface of the resin. The capacity then decreased with a further increase of Ag loading to 4.3, possibly because of the formation of large Ag_2_O particles with lower surface area in the excess of Ag content.

In order to test the potential of resin@P-Ag_2_O for practical application, the adsorption—regeneration experiment was conducted 10 times and the result is presented in Fig. 8c. It is obvious that resin@P-Ag_2_O material has a high durability with stable chloride removal after at least 10 cycles of adsorption—regeneration, which is suitable for practical application.

### Application of resin@P-Ag_2_O for COD determination of salinity water

**Figure 9:**
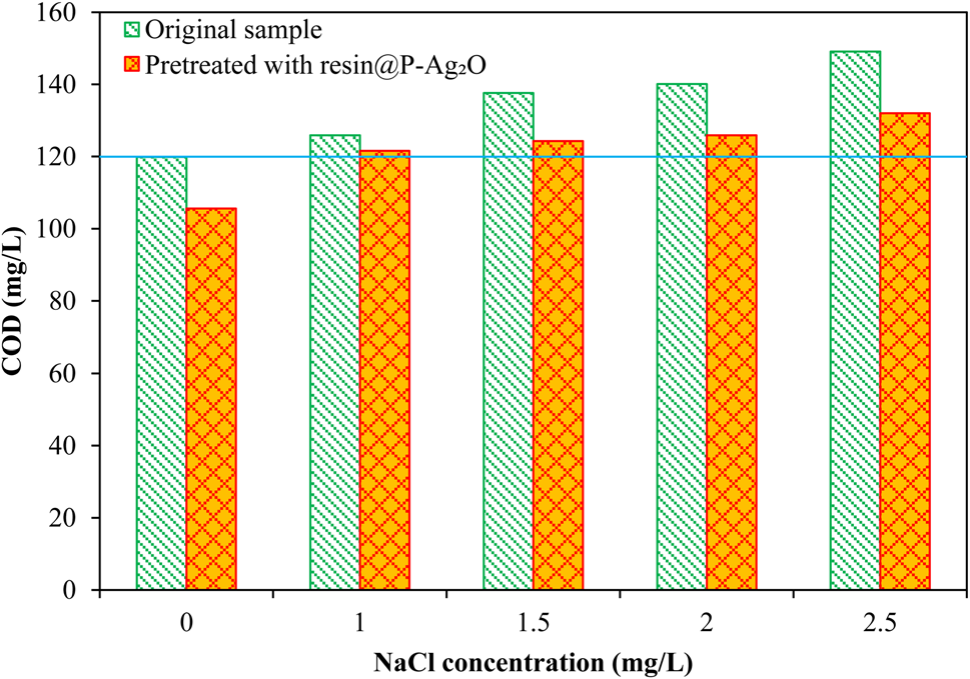
Effect of NaCl concentration to COD value of wastewater with and without using Ag_2_O/cation resin material to remove the chloride.

**Figure 10:**
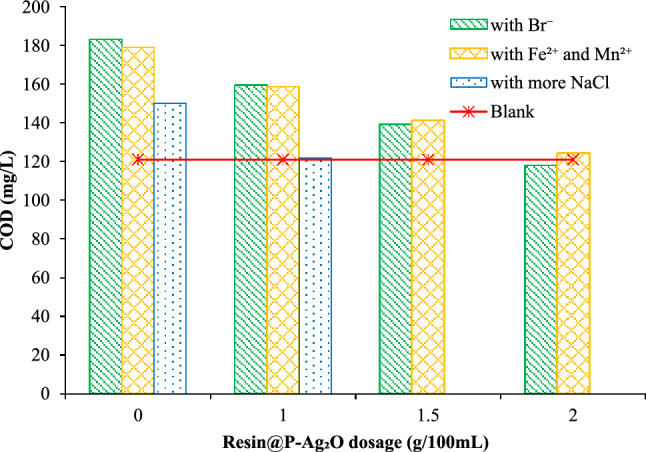
Effect of resin@P-Ag_2_O dosage on the COD measurement of 120 mgO_2_/L KHP standard solution: blank (without any salt and resin@P-Ag_2_O), with Br^−^ (1000 mg NaCl/L + 1000 mg NaBr/L), with Fe^2+^ and Mn^2+^ (1000 mg NaCl/L + 500 mgFeCl_2_/L + 500 mgMnCl_2_/L), with more NaCl (2000 mgNaCl/L).

In the environmental analysis field, COD determination of salinity water using the SMEWW 5220C:2012 method faces many difficulties due to the error from undesirable oxidation of chloride by K_2_Cr_2_O_7_ under concentrate acidic environment. Although several techniques have been proposed for improving the accuracy of the method as discussed previously, the application of these techniques is still very limited due to their own disadvantages. In this study, we proposed a new technique as an add-on but a simple step for COD measurement, and the results are displayed in Fig. 9. Results from conventional analysis (without pretreated with resin@P-Ag_2_O) showed that the COD value of solution increased with the increase of NaCl content, and therefore the COD analysis error. However, the obtained COD values for the sample pretreated with resin@P-Ag_2_O were more closed to the standard solution (without adding NaCl) with an acceptable error of ≤ 10% (positive error). For water without NaCl content, the COD value of resin@P-Ag_2_O treated sample was lower than that of the untreated one due to the adsorption of organic compounds on the resin@P-Ag_2_O, suggesting that resin@P-Ag_2_O should not be used for water sample with a salinity of < 1 g/L. For samples with salinity ≥ 1 g/L, this adsorption effect was reduced, possibly because of the predominant adsorption of chloride and the formation of AgCl on the resin. Interestingly, the COD value of sample containing 2.5 g/L of NaCl after treated with resin@P-Ag_2_O in combination with dilution method (in an analysis lab with Vilas certification) was 102 mg/L with an error of 15% (negative error, as compared to standard sample), which is probably from the dilution for reducing the salinity of water sample. These results indicate that the removal of chloride by resin@P-Ag_2_O before COD analysis is a reliable method for COD determination of salinity water samples. The effects of other ions such as iron, manganese, and bromide on the COD measurement are presented in Fig. 10, where the effects of resin@P-Ag_2_O dosage on corrected COD measurement are also provided. The effects of 1000 mgNaCl/L with 1000 mgNaBr/L or with 500 mgFeCl_2_/L + 500 mgMnCl_2_/L can be suppressed by using of 2 g of resin@P-Ag_2_O for 100 mL of water sample before doing the COD measurement. For water samples with high COD (e.g. 1000 mg/L) and chloride (e.g. 3000 mgNaCl/L), the results in Fig. S1 of Supplementary Information show that the COD could be measured with acceptable accuracy by choosing an appropriate dosage of resin@P-Ag_2_O. Although further intensive investigations are needed, this study suggests a way of using resin@P-Ag_2_O for improving the accuracy of COD measurement of salinity water, as presented in Table 1 for the suitable dosage of material for water sample with different salinities.

**Table 1:** Proposed resin@P-Ag_2_O amount for COD determination of salinity water.

Salinity (g/L or ‰)	Resin@P-Ag_2_O dosage (g/100 mL)
0–1	Do not need
1–2	1
2–4	1.5
≥ 4	Need experiments to determine

## Conclusion

Engineered resin@P-Ag_2_O material with strong acidic ion exchange resin core and Ag_2_O shell was successfully synthesized and applied for removal of chloride in water. This novel material had high chloride adsorption capacity of 244 mgCl/gAg with high durability during cycling tests. The application of this material for improving the analysis accuracy of COD for salinity water is very potential and promising with a low error of ≤ 10%. The use of resin@P-Ag_2_O in terms of dosage was also proposed base on the salinity of the water sample. This study opens a new way in the application of nanomaterial for chloride adsorption to improve the accuracy of COD measurement for high salinity water and wastewater.

## Supplementary Information

Supplementary Information.
