# Supplementary material for: Synthesis of a novel porous Ag2O nanomaterial on ion exchange resin and its application for COD determination of high salinity water
Source: Sci Rep. 2021 Jun 1;11:11487. doi: 10.1038/s41598-021-91004-w (PMC8169930; doi:10.1038/s41598-021-91004-w)
Supplement: Supplementary file 1 — Supplementary Information. [file 41598_2021_91004_MOESM1_ESM.docx]

**Supplementary information**

Table S1. Characteristic of cation exchange resin (Purolite C145)

| Polymer Structure | Macroporous polystyrene crosslinked with divinylbenzene |
| --- | --- |
| Functional group | Sulfonic Acid |
| Ionic form | Na^+^ form |
| Total capacity | 1.5 eq/L |
| Particle size range | 300 - 1200 µm |
| Moisture content | 55 - 60 % (Na^+^ form) |

Fig. S1. Effect of resin@P-Ag_2_O dosage on the COD measurement of 1000 mgO_2_/L KHP standard solution: blank (without any salt and resin@P-Ag_2_O), with NaCl and resin@P-Ag_2_O (with 3000 mg NaCl/L)
